# Additional blood meals increase mosquito infectivity by accelerating the transcriptional development of *Plasmodium* sporozoites

**DOI:** 10.64898/2026.09.26.754681

**Published:** 2026-09-28

**Authors:** W. Robert Shaw, Philipp Schwabl, Maurice A. Itoe, Shriya Anandjee, Jamie Kauffman, Lisa H. Verzier, Yan Yan, Daniel E. Neafsey, Flaminia Catteruccia

## Abstract

Sporozoites, the infectious stage of *Plasmodium* malaria parasites, must invade two different tissues in two very different hosts to ensure transmission: the mosquito salivary glands and the human liver. Whether infectivity to hepatocytes is affected by physiological components, such as the availability of nutrients within the *Anopheles* female, is not well understood. Here we show that *Plasmodium falciparum* sporozoite infectivity to hepatocytes is increased if mosquitoes take an additional blood meal while oocysts are developing. Infection rates of HC04.J7 hepatocytes at 24 h were 1.8-fold higher for *P. falciparum* salivary gland sporozoites isolated from *Anopheles gambiae* females fed twice (2BF) compared to those which only received the infectious blood meal (1BF). This increase in infectivity was observed at an early infection timepoint (day 11) and was accompanied by a marked shift in the transcriptional profiles of 2BF salivary gland sporozoites, identifying genes likely involved in infection and development in the mammalian host. Overall, our data reveal new factors potentially involved in sporozoite infectivity, and have broad implications for understanding transmission of malaria in endemic areas where multiple blood meals commonly shape available resources for adult female mosquitoes.

## Full Text Availability

The license terms selected by the author(s) for this preprint version do not permit archiving in PMC. The full text is available from the preprint server.

